# Low GSTM3 expression is associated with poor disease‐free survival in resected esophageal squamous cell carcinoma

**DOI:** 10.1186/s13000-021-01069-4

**Published:** 2021-01-22

**Authors:** Fu Yang, Jing Wen, Kongjia Luo, Jianhua Fu

**Affiliations:** 1 Department of Thoracic Surgery, Shanghai Jiaotong University First People’s Hospital, 200080 Shanghai, People’s Republic of China; 2 State Key Laboratory of Oncology in South China, Sun Yat-sen University Cancer Center, 510060 Guangzhou, People’s Republic of China; 3 Guangdong Esophageal Cancer Research Institute, 510060 Guangzhou, People’s Republic of China; 4 Department of Thoracic Oncology, Sun Yat-sen University Cancer Center, 510060 Guangzhou, People’s Republic of China

**Keywords:** Glutathione S-transferase mu 3, Esophageal squamous cell carcinoma, Prognosis, Disease‐free survival, Biomarker

## Abstract

**Background:**

Glutathione S-transferase mu 3 (GSTM3) plays a crucial role in tumor progression in various cancers. However, the relationship between GSTM3 expression and the clinical prognosis of esophageal squamous cell carcinoma (ESCC) has not been studied to date. We aimed to characterize the role of GSTM3 in predicting postoperative prognosis of ESCC patients.

**Methods:**

In the retrospective study, GSTM3 mRNA levels in 184 ESCC tissues and matched 43 adjacent nontumorous tissues were measured by quantitative real-time PCR. GSTM3 protein levels in 247 ESCC tissues were measured by immunohistochemistry.

**Results:**

Downregulation of GSTM3 occurred in 62.8 % of primary ESCC tissues compared with their nontumor counterparts. Patients with low GSTM3 expression tended to exhibit an increased rate of poor differentiation in both the mRNA cohort (*p* = 0.024) and protein cohort (*p* = 0.004). In the mRNA cohort, low GSTM3 expression was associated with unfavorable 3-year disease-free survival (DFS) (39.2 % vs. 57.4 %) and 5-year DFS (26.8 % vs. 45.1 %) (*p* = 0.023). The result was confirmed in the protein cohort. Patients with low GSTM3 expression had unfavorable 3-year disease-free survival (DFS) (18.7 % vs. 33.5 %) and 5-year DFS (5.3 % vs. 30.5 %) (*p* = 0.006). Cox multivariate analysis revealed that GSTM3 expression was an independent prognostic factor.

**Conclusions:**

The findings of the present study provide evidence that GSTM3 may function as a tumor suppressor in ESCC and represents a potential novel prognostic biomarker for disease-free survival for resected ESCC patients.

## Background

Esophageal cancer (EC) has been ranked as the eighth most common malignancy and the sixth most common cause of cancer deaths worldwide [1]. The incidence of esophageal cancer varies greatly by geographic location and ethnicity, with a 60-fold difference between high- and low-incidence regions [2]. Esophageal cancer has 2 major histologic types: squamous cell carcinoma (SCC) and adenocarcinoma (ACE) [3]. Esophageal squamous cell carcinoma (ESCC) is the most common histology in Asia and Eastern Europe, accounting for greater than 90 % of all EC in China [4, 5]. Tobacco and alcohol abuse, environmental carcinogens, and occupational exposure are major risk factors for SCC of the esophagus [6, 7]. Patients with ESCC are also at increased risk of developing second primary cancers, such as head and neck tumors and lung cancer [8]. This feature suggests that the oxidation-reduction system of patients with ESCC may be impaired. Carcinogens are one of the inducing etiological factors for esophageal cancer. Glutathione S-transferases (GST), a supergene family of enzymes involved in phase II detoxification of toxins and enzymes, play important roles in the prevention of cancer by detoxifying numerous potentially carcinogenic compounds [9, 10]. Therefore, GST deficiencies may increase the risk of carcinogenesis. At least five mammalian GST gene families have been identified as polymorphic, and mutations or deletions of these genes contribute to the predisposition of several diseases, including cancer. The gene cluster of GSTM1-GSTM5 is located on chromosome 1p13. The correlation between GST enzyme activity and tumor incidence has been demonstrated in the esophageal squamous cell carcinoma, esophageal adenocarcinoma, gastric cancer and colorectal cancer [11–15].

GSTM3 is one of the GST mu-classes that plays a role in the metabolism of harmful agents, such as polyaromatic hydrocarbons benzo(α)pyrene, and exhibits overlapping substrate specificity with GSTM1 [9]. Low GSTM3 expression in ESCC compared to adjacent benign epithelial was identified in our previous study based on DNA microarray analysis [16]. GSTM3 polymorphisms may increase lung cancer and esophageal cancer susceptibility [11, 17]. Nonetheless, the relationship between GSTM3 expressions and ESCC requires further elucidation. Therefore, the aim of the present study is to verify GSTM3 expression in primary ESCC and analyze its correlation with clinical parameters. In the present study, GSTM3 mRNA expression was assessed in primary ESCC tissues from 184 patients collected immediately after surgical resection, and GSTM3 protein levels tested in ESCC tissue microarrays. We correlated GSTM3 expression with clinical and pathologic features, including survival outcomes. Our findings indicate that low GSTM3 expression is predictive of poorer disease-free survival for patients with resected ESCC. These data provide evidence that GSTM3 could serve as a biomarker of ESCC prognosis.

## Materials and methods

### Patients and tissue samples

Primary ESCC tissues from one hundred and eighty-four patients and forty-three paired adjacent nontumorous tissues were collected immediately after surgery resection at Sun Yat-sen University Cancer Center from March 2002 to October 2008. The inclusion criteria were as follows: histological proof of thoracic ESCC, complete surgical resection (R0), no neoadjuvant or adjuvant treatment and complete follow-up data. The study was approved by the Ethics Committee of Sun Yat-sen University Cancer Center. All the patients signed informed consent.

### Quantitative real‐time Polymerase Chain Reaction (qPCR)

Fresh tumorous and nontumorous samples were obtained from regions that were macroscopically judged to be neoplastic and normal, respectively. The samples were immediately stored in dry ice after resection and then frozen at -80°C. Total RNA was extracted from clinical samples using TRIzol reagent (Invitrogen) according to the manufacturer’s instruction. cDNA was synthesized from 1 µg of total RNA using RevertAid First Strand cDNA Synthesis Kit (Thermo Scientific) and stored at -80°C. cDNA was subjected to quantitative real-time PCR (qRT-PCT) for GSTM3. GAPDH was used as an internal control for GSTM3. The primers for GSTM3 and GAPDH are shown in Table 1. qRT-PCR was performed using the Power SYBR Green PCR Master Mix (Applied Bio systems) and LightCycler480 384-well PCR system (Roche Diagnostics). RT-PCR was performed using the following thermal cycling profile: denaturing step at 95°C for 10 min; 40 cycles of amplification (95°C for 10 s, 60°C for 20 s); and then 72°C for 30 s. The assays were performed in triplicate, and values were normalized using the internal control. PCR products were subjected to dissociation curve analysis to exclude amplification of nonspecific products. The value of relative expression was calculated using the 2-△△Ct method. △△Ct (sample)= △Ct (sample)- △Ct (calibrator); △Ct (sample) = Ct (sample) of GSTM3-Ct (sample) of GAPDH; △Ct (calibrator) = Ct (calibrator) of GSTM3-Ct (calibrator) of GAPDH. The calibrator was defined as the pooled adjacent nontumor tissue samples from 43 patients.

**Table 1:** Primer sequences used for qPCR analyses

Gene		Sequence (5’->3’)	Accession number
GSTM3	Forward sequence	CCAATGGCTGGATGTGAA	NM_000849.5
	Reverse sequence	GGTAGGGCAGATTAGGAAAGT	
GAPDH	Forward sequence	ACTTCAACAGCGACACCCACTC	NM_001256799.1
	Reverse sequence	TACCAGGAAATGAGCTTGACAAAG	

### ESCC Tissue Microarray (TMA) and Immunohistochemical (IHC) staining

The ESCC tissue microarray (TMA) with a total of 290 formalin-fixed, paraffin-embedded ESCC tumor specimens and the corresponding normal epithelia was kindly provided by Prof. Xinyuan Guan from State Key Laboratory of Oncology in Southern China, Sun Yat-sen University Cancer Center. TMA were constructed using a Beecher Instruments tissue microarrayer (Beecher Instruments, Sun Prairie, WI). Three targeted core samples with a 1-mm diameter of each specimen were punched and arrayed on a recipient paraffin block to construct the tissue microarray. For immunohistochemical (IHC) analysis, the slides were deparaffinized, rehydrated, and blocked by 5 % normal goat serum at room temperature for 30 min. The slides were then incubated with mouse anti-GSTM3 antibody (Developmental Studies Hybridoma Bank) at a dilution of 1:100 at 4°C overnight and subsequently incubated with biotinylated goat anti-mouse immunoglobulin at a concentration of 1:100 for 30 min at 37°C. The ESCC tissue microarrays were scored semiquantitatively on the basis of a well-established immunoreactivity scoring system (IRS) as described previously [18]. The IRS was calculated by the intensity of GSTM3-positive staining (0, no staining; 1, mild; 2, moderate; 3, strong) and the percentage of GSTM3-positive cells (0, ≤ 5 %; 1, 6–25 %; 2, 26–50 %; 3, 51–75 %; 4, > 75 %). The total score was determined by the following formula: Staining index = intensity × positive rate. IRS scores range between 0 (no staining) and 12 (maximum staining).

Two independent observers blinded to the clinic pathologic information performed the evaluation of GSTM3 expression. If the two observers conflicted with each other, a third independent observer was asked to determine the final result.

### Statistical methods

Receiver operative characteristic (ROS) curve generated by MedCalc 15.2.2 (MedCalc Software, Mariakerke, Belgium) was used to determine the cutoff value for GSTM3 mRNA expression that yielded the highest combined sensitivity and specificity with respect to distinguishing disease-specific 5-year survivors from nonsurvivors. Statistical analysis was performed using the Statistical Package for the Social Sciences (SPSS) version 16.0 for windows software system (SPSS Inc., Chicago, IL). Paired t test was employed to compare the expression of GSTM3 mRNA in primary ESCC tumor tissues and corresponding adjacent nontumorous tissues. The correlation between GSTM3 expression and clinicopathologic characteristics was assessed by χ2 or Fisher’s exact tests. Disease-specific survival (DSS) was calculated from the time of surgery to either the time of death from ESCC or last follow-up. To the time of last follow-up or death from disease other than ESCC, at which point, the data were censored. The prognostic value of GSTM3 expression for predicting survival was calculated using the Kaplan-Meier method and analyzed by log-rank test. Univariate survival analysis was performed using the Cox’s proportional hazard modes. To determine independent factors that were significantly related to the prognosis, multivariate analysis was performed using a Cox’s proportional hazard regression model with a forward stepwise procedure (the entry and removal probabilities were 0.05 and 0.10, respectively). A significant difference was declared if the pvalue from a two-tailed test was less than 0.05.

## Results

### Quantitative real‐time Polymerase Chain reaction (qRT-PCR) assays

**Fig. 1:**
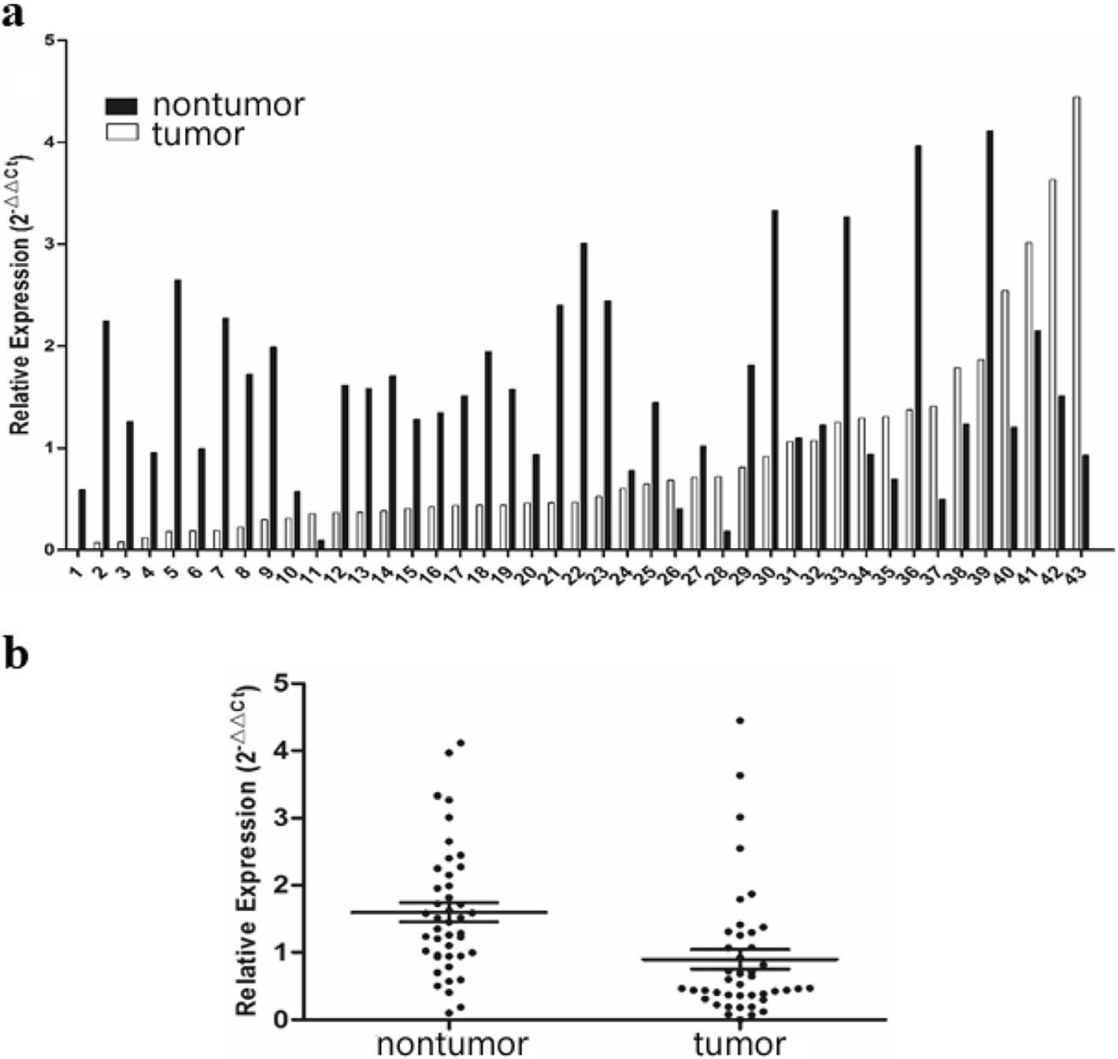
GSTM3 was downregulated in esophageal squamous cell carcinoma. **a** and **b**: GSTM3 mRNA was markedly decreased in tumor tissues compared with paired adjacent nontumor tissues (*p* = 0.001, paired t-test)

**Fig. 2:**
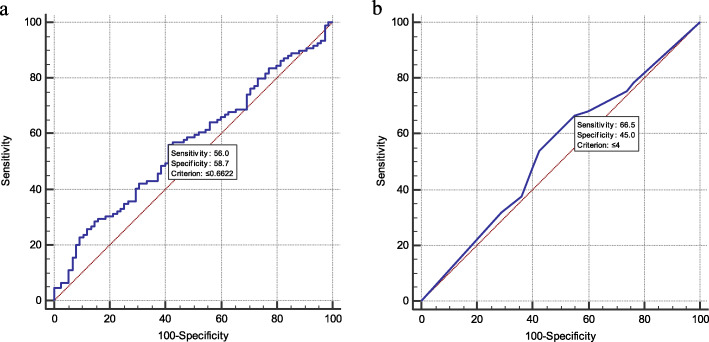
Receiver operating characteristic (ROC) curve. **a** ROC curve based on GSTM3 mRNA expression levels. The optimal cut-off value was 0.662 with a sensitivity of 56.0 % and a specificity of 58.7 %. The area under the ROC curve was 0.564, and the *p*-value was 0.135. **b** ROC curve based on GSTM3 protein expression. The optimal cut-off value was 4, with a sensitivity of 66.5 % and a specificity of 45.0 %. The area under the ROC curve was 0.539, and the *p*-value was 0.311

GTM3 was frequently downregulated in ESCC. GSTM3 mRNA expression was initially tested in 43 pairs of primary ESCC tumors and their adjacent nontumorous tissues by qPCR. Reduced GSTM3 expression was detected in 27 of 43 (62.8 %) of ESCC tumors compared with paired adjacent nontumorous tissues (defined as a 2-fold decrease in GSTM3 expression in tumors) (Fig. 1a). The relative expression level of GSTM3 was significantly downregulated in tumor tissues compared with paired adjacent nontumorous tissues (*p* = 0.001, Fig. 1b). In the mRNA cohort, which includes 184 samples, the optimal cutoff value of GSTM3 was 0.662 based on the ROC curve (Fig. 2a). At this threshold of GSTM3, the sensitivity was 56.0 %, and the specificity was 58.7 %. Then, GSTM3 mRNA expression in the mRNA cohort was divided into two groups: the low-expression group (≤ 0.662, *n* = 91) and the high-expression group (>0.662, *n* = 93).

### ESCC tissue microarray and immunohistochemical staining

**Fig. 3:**
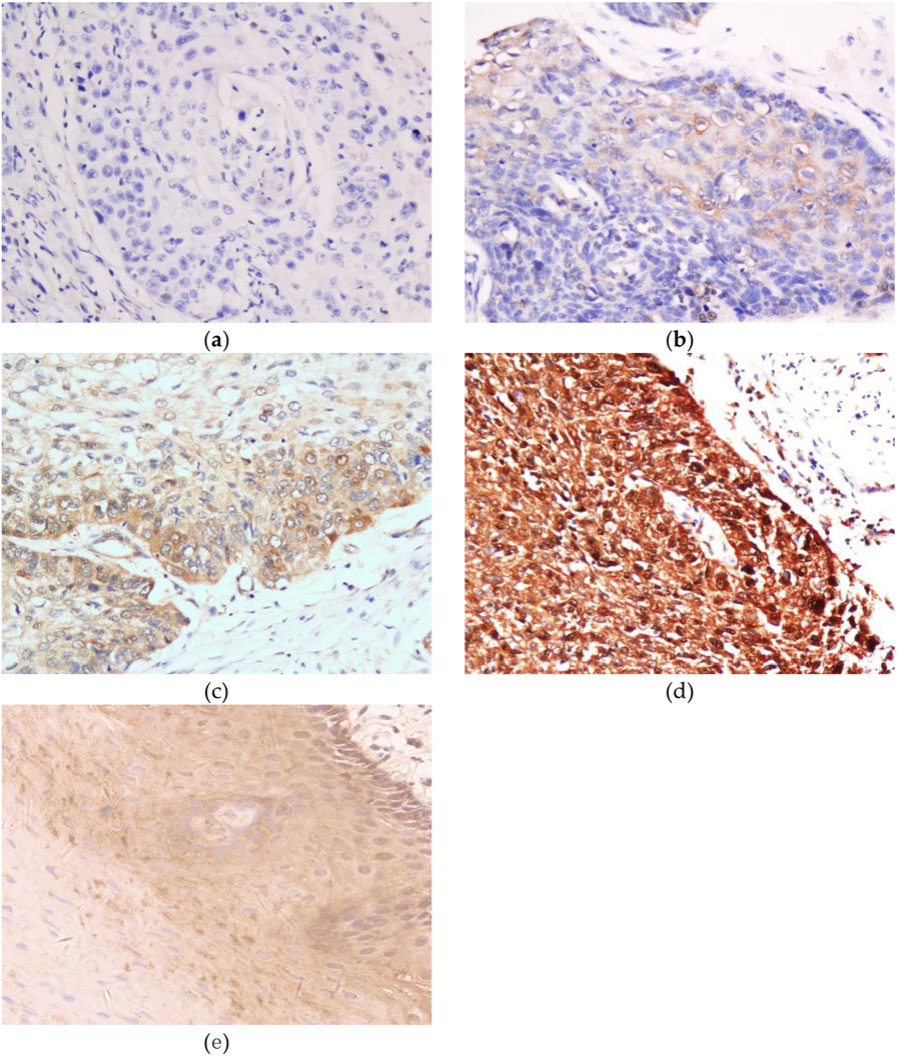
Representative images of GSTM3 expression in ESCC tumor tissues and adjacent nontumorous tissue detected by immunostaining with anti-GSTM3 antibody. **a**: negative; **b**: weakly positive; **c**: medium positive; **d**: strong positive; **e**: adjacent nontumorous tissue, medium positive. The slide was counterstained with hematoxylin (original magnification × 200)

GSTM3 expression at the protein level was further studied in 290 primary ESCCs by immunohistochemical (IHC) using tissue microarray (TMA) (Fig. 3). Informative IHC results were obtained from 247 pairs of ESCCs. Noninformative samples included lost samples, unrepresentative samples, and samples with too few tumor cells. Receiver operative characteristic (ROC) curve generated by MedCalc 15.2.2 (MedCalc Software, Mariakerke, Belgium) was used to determine the cutoff value for GSTM3 protein expression. According the ROC curve (Fig. 2b), the optimal cutoff value of GSTM3 with the best discriminatory power was determined to be 4. The staining index of GSTM3 in each informative tumor tissue that was greater than or equal to 6 was classified as high expression, whereas an index of 0–4 indicated low expression. Using this designation, high GSTM3 expression was detected in 155 of 247 (62.75 %) ESCC tissues, whereas 92 of 247 (37.25 %) informative ESCC tissues were classified as low expression.

### Clinicopathologic features of GSTM3 in ESCC patients

In total, 184 patients with primary ESCCs (mRNA cohort) and an additional 247 patients with primary ESCCs (protein cohort) were recruited in the study. Follow-up data were obtained from all patients with a median survival of 41 months in the mRNA cohort (range, 2-115 months) and 23 months in the protein cohort (range, 2–60 months). According to the 8th edition AJCC staging system [19] and our demographic data, the clinicopathologic features were dichotomized for statistical analyses as shown in Table 2. GSTM3 expression was significantly associated with histological differentiation of ESCC (*p* = 0.024 in the mRNA cohort and 0.004 in the protein cohort). Patients with low GSTM3 expression tended to have a higher rate of poor differentiation in both the mRNA cohort and protein cohort. No significant association was observed between GSTM3 expression and patient’s age, gender, tumor location, pT category, pN category and pathological stage in the mRNA cohort and the protein cohort.

**Table 2:** The association between GSTM3 expression and clinicopathologic features in patients with ESCC

Characteristic	GSTM3 expression in mRNA cohort			P^#^	GSTM3 expression in protein cohort			P^#^
	**Case**	**Low level (%)**	**High level (%)**		**Case**	**Low level (%)**	**High level (%)**	
Gender				0.618				0.691
Male	135	65 (48.1)	70 (51.9)		137	84 (61.3)	53 (38.7)	
Female	49	26 (53.1)	23 (46.9)		110	71 (64.5)	39 (35.5)	
Age				1.000				0.356
≤ 60	104	51 (49.0)	53 (51.0)		138	83 (60.1)	55 (39.9)	
> 60	80	40 (50.0)	40 (50.0)		109	72 (66.1)	37 (33.9)	
Location				0.543				0.567
Upper	40	19 (47.5)	21 (52.5)		60	35 (58.3)	25 (41.7)	
Middle	100	47 (47.0)	53 (53.0)		161	105 (65.2)	56 (34.8)	
Lower	44	25 (56.8)	19 (43.2)		26	15 (57.7)	11 (42.3)	
Differentiation				**0.024**				**0.004**
Grade 1	46	17 (37.0)	29 (63.0)		23	12 (52.2)	11 (47.8)	
Grade 2	92	44 (47.8)	48 (52.2)		157	90 (57.3)	67 (42.7)	
Grade 3	46	30 (65.2)	16 (34.8)		67	53 (79.1)	14 (20.9)	
pT category				0.084				1.000
T1-2	44	27 (61.4)	17 (38.6)		84	53 (63.1)	31 (36.9)	
T3-T4	140	64 (45.7)	76 (54.3)		163	102 (62.6)	61 (37.4)	
pN category				0.660				0.356
N0	100	51 (51.0)	49 (49.0)		136	89 (65.4)	47 (34.6)	
N1-3	84	40 (47.6)	44 (52.4)		111	66 (59.5)	45 (40.5)	
Pathological staging				0.371				0.506
I	14	9 (64.3)	5 (35.7)		19	13 (68.4)	6 (31.6)	
II	82	42 (51.2)	40 (48.8)		119	78 (65.5)	41 (34.5)	
III-IV	88	40 (45.5)	48 (54.5)		109	64 (58.7)	45 (41.3)	

^#^Chi-square test

### Association between GSTM3 expression and patient survival

**Fig. 4:**
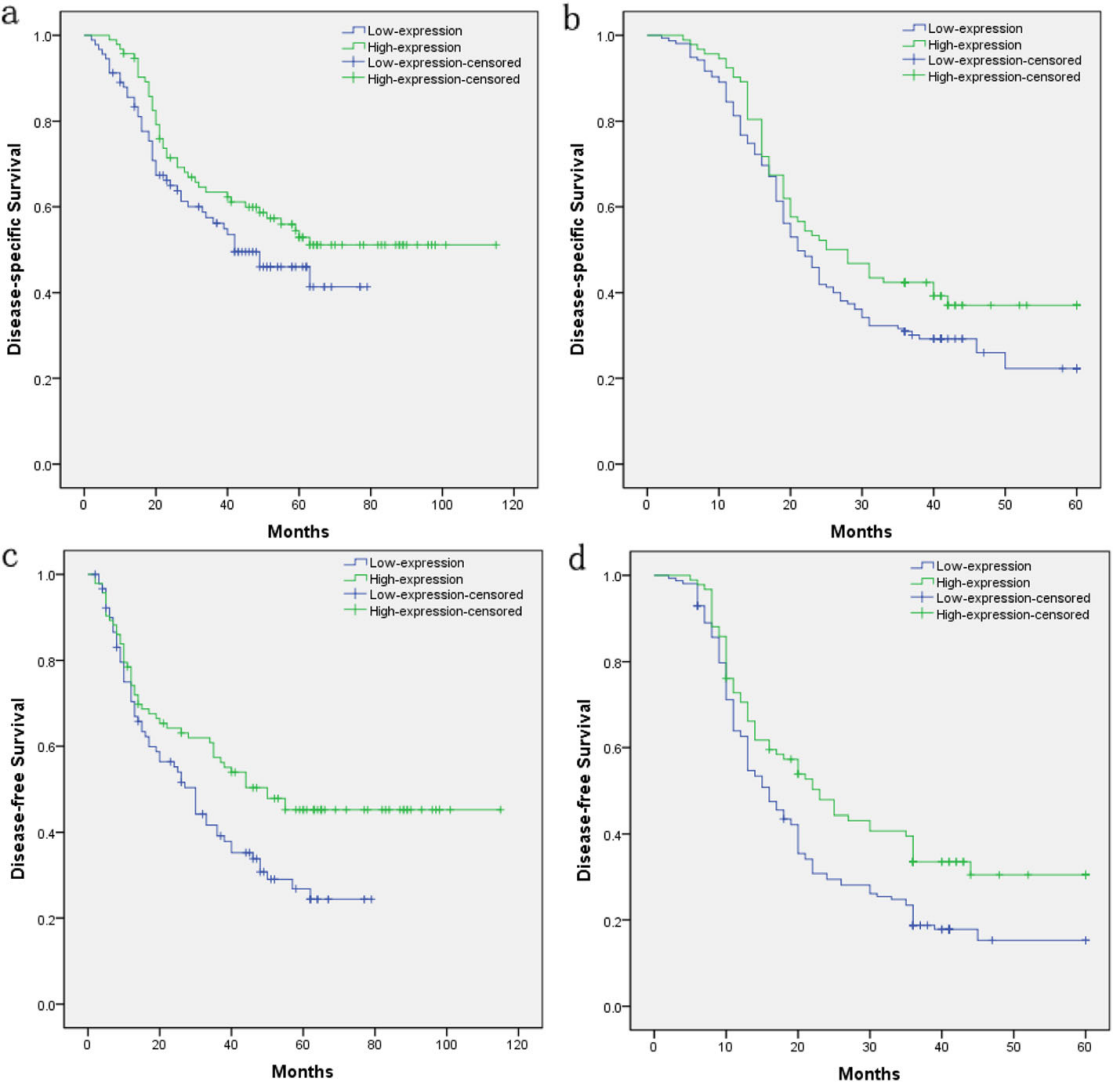
Kaplan-Meier survival plots for disease-specific survival and disease-free survival based on low expression of GSTM3 versus high expression of GSTM3 for ESCC patients. **a** Disease-specific survival for ESCC patients in the mRNA cohort. No significant difference in DSS between low-expression and high-expression groups, log-rank test, *p* = 0.132. **b** Disease-specific survival for ESCC patients in the protein cohort. No significant difference in DSS between low-expression and high-expression groups, log-rank test, *p* = 0.070. **c** Disease-free survival for ESCC patients in the mRNA cohort. Patients with low GSTM3 expression had a poorer disease-free survival than those with high GSTM3 expression, log-rank test, *p* = 0.016. **d** Disease-free survival for ESCC patients in the protein cohort. Patients with low GSTM3 expression exhibited poorer disease-free survival compared with those with high GSTM3 expression, log-rank test, *p* = 0.006

Kaplan-Meier analysis revealed no significant difference in DSS between low- and high-level expression groups in both the mRNA cohort and protein cohort of ESCC patients (Fig. 4a and b). Kaplan-Meier analysis showed that low GSTM3 expression was significantly associated with poorer disease-free survival (DFS) of resected ESCC patients in both the mRNA cohort and protein cohort (Fig. 4c and d). The 3-year DFS and 5-year DFS of ESCC in low- and high-level expression groups in the mRNA cohort were 39.2 % vs. 57.4 % and 26.8 % vs. 45.1 %, respectively (*p* = 0.016) (Table 3). The 3-year DFS and 5-year DFS of ESCC in low- and high-level expression groups in the protein cohort were 18.7 % vs. 33.5 % and 15.3 % vs. 30.5 %, respectively (*p* = 0.006) (Table 4). Cox’s proportional hazards regression confirmed that high GSTM3 expression was significantly associated with lower risk of disease recurrence in the mRNA cohort (hazard ratio, HR: 0.635, 95 % confidence interval, CI: 0.435–0.927, *p* = 0.019) and protein cohort (HR: 0.659, 95 % CI: 0.484–0.898, *p* = 0.008) (Tables 5 and 6).

**Table 3:** Univariate analysis of GSTM3 expression and clinicopathological factors for DSS and DFS in mRNA cohort patients with ESCC

Characteristic	Cases	Disease-specific survival (DSS) (%)		P^#^	Disease-free survival (DFS) (%)		P^#^
		**3-year DSS**	**5-year DSS**		**3-year DFS**	**5-year DFS**	
Gender				0.450			0.830
Male	135	60.6	50.8		49.2	37.1	
Female	49	58.2	44.5		46.7	34.9	
Age				0.096			0.382
≤ 60	104	64.5	53.6		53.5	40.6	
> 60	80	54.3	43.8		42.3	31.3	
Location				0.228			0.500
Upper	40	53.9	40.0		44.0	34.9	
Middle	100	59.8	50.7		48.2	34.4	
Lower	44	65.6	52.3		53.4	43.1	
Differentiation				0.217			**0.048**
Grade 1	46	68.8	59.6		61.6	49.9	
Grade 2	92	61.5	47.8		47.4	33.2	
Grade 3	46	47.2	41.5		37.5	30.0	
pT category				0.064			0.098
T1-2	44	67.8	61.4		57.0	48.3	
T3-4	140	57.4	45.4		45.9	32.9	
pN category				**0.000**			**0.000**
N0	100	76.9	65.7		69.2	53.6	
N1-3	84	40.0	30.0		25.0	17.3	
Pathological staging				**0.000**			**0.000**
I	13	92.3	80.8		92.3	71.2	
II	92	72.3	61.8		62.8	50.1	
III	79	40.2	29.6		25.4	15.8	
GSTM3 expression				0.132			**0.016**
Low	91	56.2	46.0		39.2	26.8	
High	93	64.6	52.8		57.4	45.1	

^#^Kaplan-Meier method (log-rank test)

**Table 4:** Univariate analysis of GSTM3 expression and clinicopathological factors for DSS and DFS in protein cohort patients with ESCC

Characteristic	Cases	Disease-specific survival (DSS) (%)		***P***^**#**^	Disease-free survival (DFS) (%)		***P***^**#**^
		3-year DSS	5-year DSS		3-year DFS	5-year DFS	
Gender				0.536			0.188
Male	137	31.4	24.4		19.3	18.2	
Female	110	40.0	32.4		30.3	25.0	
Age				0.210			0.214
≤60	138	37.7	32.8		31.9	23.3	
>60	109	32.1	21.5		22.0	17.7	
Location				0.148			0.054
Upper	60	25.0	20.8		12.5	12.5	
Middle	161	37.9	29.3		26.3	21.3	
Lower	26	42.3	35.3		37.5	37.5	
Differentiation				0.098			**0.046**
Grade 1	23	52.2	52.2		43.5	36.2	
Grade 2	157	35.0	24.3		24.7	23.7	
Grade 3	67	29.9	27.9		15.8	7.9	
pT category				**0.001**			**0.017**
T1-2	84	48.8	42.0		29.7	28.3	
T3-4	163	28.2	21.5		21.2	15.9	
pN category				**0.000**			**0.002**
N0	136	45.6	35.2		29.3	24.1	
N1	111	22.5	19.7		17.7	17.7	
Pathological staging				**0.000**			**0.001**
I	13	69.2	69.2		38.5	38.5	
II	149	43.0	32.7		27.8	23.3	
III	85	16.5	16.5		15.4	15.4	
GSTM3 expression				0.070			**0.006**
Low	155	31.0	22.3		18.7	15.3	
High	92	42.4	37.1		33.5	30.5	

^#^Kaplan-Meier method (log-rank test)

**Table 5:** Univariate analysis of prognostic variables^a^ for DSS and DFS in mRNA cohort patients with ESCC

Characteristic	Disease-specific survival (DSS)		Disease-free survival (DFS)	
	HR (95% CI)	P	HR (95% CI)	P
Gender (female vs. male)	1.188 (0.752-1.879)	0.460	1.046 (0.688-1.592)	0.832
Age (>60 vs. ≤60)	1.420 (0.935-2.157)	0.100	1.179 (0.810-1.716)	0.389
Location (lower vs. middle vs. upper)	0.772 (0.563-1.058)	0.108	0.862 (0.653-1.138)	0.295
Differentiation (G3 vs. G2 vs. G1)	1.303 (0.965-1.751)	0.084	1.375 (1.054-1.793)	0.019
pT category (T3-4 vs. T1-2)	1.555 (0.974-2.482)	0.064	1.420 (0.958-2.105)	0.081
pN category (N1-3 vs. N0)	2.992 (1.928-4.641)	0.000	3.146 (2.129-4.649)	0.000
Pathological staging (III-IV vs. II vs. I)	2.497 (1.678-3.717)	0.000	2.573 (1.810-3.657)	0.000
GSTM3 expression (high vs. low)	0.727 (0.477-1.107)	0.137	0.635 (0.435-0.927)	0.019

^a^Cox’s proportional hazards regression analysis (forward stepwise)

*HR* hazard risk, *CI* confidence interval, *G1* grade 1, *G2* grade 2, *G3* grade 3

**Table 6:** Univariate analysis of prognostic variables^a^ for DSS and DFS in protein cohort patients with ESCC

Characteristic	Disease-specific survival (DSS)		Disease-free survival (DFS)	
	HR (95% CI)	***P***	HR (95% CI)	***P***
Gender (female vs. male)	0.909 (0.668-1.237)	0.544	0.826 (0.616-1.108)	0.826
Age (>60 vs. ≤60)	1.210 (0.892-1.641)	0.220	1.194 (0.894-1.595)	0.230
Location (lower vs. middle vs. upper)	0.782 (0.598-1.022)	0.071	0.742 (0.575-0.957)	0.021
Differentiation (G3 vs. G2 vs. G1)	1.324 (1.015-1.727)	0.038	1.358 (1.057-1.745)	0.017
pT category (T3-4 vs. T1-2)	1.738 (1.233-2.450)	0.002	1.441 (1.056-1.966)	0.021
pN category (N1-3 vs. N0)	1.936 (1.425-2.628)	0.000	1.566 (1.171-2.093)	0.003
Pathological staging (III-IV vs. II vs. I)	1.985 (1.526-2.582)	0.000	1.581 (1.243-2.011)	0.000
GSTM3 expression (high vs. low)	0.748 (0.542-1.032)	0.077	0.659 (0.484-0.898)	0.008

^a^Cox’s proportional hazards regression analysis (forward stepwise)

*HR* hazard risk, *CI* confidence interval, *G1* grade 1, *G2* grade 2, *G3* grade 3

Univariate analysis presented in Table 3 demonstrated that pN category and pathological staging were closely associated with 3- and 5-year DSS and DFS in the mRNA cohort. Patients with T1-2 tended to have better DSS and DFS compared with patients with T3-4 in the mRNA cohort; however, the result was not statistically significant. In the protein cohort, univariate analysis showed that pT category, pathological nodal status and staging were also significant prognostic factors of DSS and DFS (Table 4). Histological differentiation was significantly associated with DFS in both the mRNA and protein cohort.

Further multivariate survival analysis showed that pN category was an independent prognostic factor for disease-specific survival in the mRNA cohort, and pT category and pN category were independent prognostic factors for disease-specific survival in the protein cohort (Table 7). Another multivariate analysis was conducted including the tumor location, histological differentiation, pathologic-T category, N status, and GSTM3 expression to evaluate the independent prognostic significance for disease-free survival in both cohorts (Table 8). Cox’s proportional hazards regression indicated that pN category and GSTM3 expression were independent prognostic factors for disease-free survival in the mRNA cohort, and tumor location, pN category and GSTM3 expression were independent prognostic factors for disease-free survival in the protein cohort. High GSTM3 expression levels indeed decreased the risk of disease recurrence for patients with resected ESCC compared with those with low GSTM3 expression levels. The hazard ratios were 0.629 (95 % CI: 0.425–0.931, *p* = 0.021) and 0.636 (95 % CI: 0.464–0.872, *p* = 0.005) for disease recurrence in the mRNA cohort and protein cohort, respectively (Table 8).

**Table 7:** Multivariate survival analysis ^a^ for disease-specific survival in patients with ESCC

Prognostic factor	mRNA cohort		Protein cohort	
	**HR (95 % CI)**	***P***	**HR (95 % CI)**	***P***
Age (>60 vs. ≤60)	1.368 (0.897–2.087)	0.146	-	-
Differentiation (G3 vs. G2 vs. G1)	-	-	1.241 (0.954–1.614)	0.107
pT category (T3-4 vs. T1-2)	1.370 (0.780–2.407)	0.273	1.525 (1.069–2.175)	0.020
pN category (N1-3 vs. N0)	2.919 (1.876–4.540)	0.000	1.775 (1.291–2.440)	0.000
GSTM3 expression (High vs. Low)	-	-	0.739 (0.533–1.024)	0.069

^a^Cox’s proportional hazards regression analysis (forward stepwise); *HR* hazard ratio, *95 % CI* 95 % confidence interval; -, unavailable

**Table 8:** Multivariate survival analysis ^a^ for disease-free survival in patients with ESCC

Prognostic factor	mRNA cohort		Protein cohort	
	HR (95% CI)	***P***	HR (95% CI)	***P***
Location (Lower vs. Middle vs. Upper)	-	-	0.673 (0.516-0.878)	0.003
Differentiation (G3 vs. G2 vs. G1)	1.149 (0.869-1.520)	0.331	1.241 (0.970-1.588)	0.085
pT category (T3-4 vs. T1-2)	1.402 (0.859-2.288)	0.177	1.344 (0.974-1.855)	0.072
pN category (N1-3 vs. N0)	2.902 (1.940-4.343)	0.000	1.608 (1.180-2.191)	0.003
GSTM3 expression (High vs. Low)	0.629 (0.425-0.931)	0.021	0.636 (0.464-0.872)	0.005

^a^Cox’s proportional hazards regression analysis (forward stepwise)

*HR* hazard ratio, *95% CI* 95% confidence interval; -, unavailable

## Discussion

Glutathione S-transferases (GSTs) are cellular phase II detoxification enzymes that catalyze the conjugation of the reduced glutathione (GSH) to endogenous and exogenous electrophilic chemicals, rendering the products more water-soluble to be eliminated from the cell. This finding suggested that GSTM3 might be associated with the prognosis of several cancers [20–23]. Glutathione S-transferase mu 3 is downregulated in ovarian cancer as demonstrated using proteomics analysis [24]. In chemical-induced hepatocarcinogenesis, a significant reduction of GSTM3 expression was observed [25]. Epigenetic inactivation of GSTM3 has been reported in Barrett’s adenocarcinoma [26]. In a study on renal cell carcinoma (RCC), GSTM3 was not only downregulated in primary RCC tissues compared with adjacent normal renal tissues but also downregulated in metastatic RCC cells compared with primary RCC cells [27]. These studies indicated that GSTM3 expression was downregulated in these cancer types. Our result is consistent with these aforementioned studies. Downregulation of GSTM3 mRNA levels was detected in 62.8 % of ESCC tumors compared with paired adjacent nontumor tissues.

However, the correlations between GSTM3 expression and clinical endpoints have rarely been assessed. The significance of GSTM3 expression levels in tumor prognosis remains inconsistent. Some studies have noted that high GSTM3 expression is a poor prognostic factor, whereas others have noted that low GSTM3 expression is a poor prognostic factor. In the study conducted by Kearns PR, lymphoblast expression of GSMT3 was positively associated with good prognosis in childhood acute lymphoblastic leukemia [20]. Meding S reported that high GSTM3 expression correlated with lymph node metastasis and advanced stage of colon cancer, and low GSTM3 expression was associated with better survival [21]. In bladder cancer, patients with low GSTM3 expression exhibited the highest survival probability, whereas whose with normal or high GSTM3 expression had lower survival probability [22]. Thus, GSTM3 function appears to be context dependent and may vary with different cancer types.

We stratified the patients into high- and low-expression level groups according to the best cutoff value determined by ROC curve generated from MedCalc with the highest combined sensitivity and specificity with respect to distinguishing 5-year survivors from nonsurvivors. We investigated the associations of GSTM3 expression levels with the clinical-pathological features of ESCC. Our data demonstrated that GSTM3 expression is related to tumor differentiation; patients with low GSTM3 expression in tumor tissue exhibit an increased rate of poor differentiation in both mRNA cohort and protein cohorts. However, no relationships were found between GSTM3 expression levels and patient’s age, gender, tumor location, tumor invasion, lymph node metastasis, and pathological stage. To validate its potential clinical utility, we evaluated the predictive power of GSTM3 at both mRNA and protein levels. Univariate analysis demonstrated that patients with high GSTM3 expression tended to have better 3- and 5-year DSS compared with those with low-expression in the mRNA cohort (64.6 % vs. 56.2 % and 52.8 % vs. 46.0 %, *p* = 0.132) and the protein cohort (42.4 % vs. 31.0 % and 37.1 % vs. 22.3 %, *p* = 0.070); however, the difference was not statistically significant. Interestingly, our data indicated that low GSTM3 expression is significantly associated with increased risk of tumor recurrence. The correlation is confirmed by immunohistochemistry in the protein cohort. These data indicate that GSTM3 may function as a tumor suppressor in ESSC. Downregulation of GSTM3 promotes disease relapse and metastasis. To the best of our knowledge, this is the first study to report that GSTM3 was a prognosis factor of ESCC.

Our present study showed that the difference of DFS between low and high expression of GSTM3 was statistically significant, but the difference in DSS did not reach statistical significance. We hypothesize that GSTM3 may be related to sensitivity to chemotherapy, radiotherapy, or chemoradiation. GSTs have been implicated in the development of resistance to chemotherapy agents [9, 28, 29]. Elevated GST levels are associated with increased resistance to apoptosis initiated by a variety of anti-cancer drugs. It is presumed that GSTs serve two distinct roles in the development of drug resistance via direct detoxification as well as acting as an inhibitor of the MAP kinase pathway [9, 30–32]. Both low- and high-expression of GSTM3 cohorts received no neoadjuvant or adjuvant treatment until disease recurrence. We could not draw a conclusion about relationship between GSTM3 expression and sensitivity to chemotherapy and radiotherapy.

Our study had several limitations. To better elucidate the role of GSTM3 in ESCC, the following challenges should be met in the future. First, our cohort study was a retrospective study, which may lead to selection bias. Second, although we demonstrated that GSTM3 was associated with the DFS of the ESCC, the role of GSTM3 in the proliferation and invasion of ESCC in vitro and vivo remains unclear. Further research on cell cycle analysis, apoptosis analysis, invasion assays and tumor formation in vivo are required to explore the tumor-suppressive ability of GSTM3 and its related pathway. Third, data about treatment after disease recurrence was absent. We could not conclude the relationship between GSTM3 expression and sensitivity to chemotherapy or radiotherapy. Further studies are required to unveil the anti-cancer drug resistance and radiation insensitivity of GSTM3 in ESCC.

In conclusion, the results of the present study for the first time demonstrated that GSTM3 may function as a tumor suppressor in ESCC. Patients with low GSTM3 expression tended to exhibit an increased rate of poor differentiation in patients with resectable ESCC. Low GSTM3 expression in ESCC tumorous specimens indicated aggressive tumor behaviors and predicted poorer disease-free survival. These findings will be helpful in the surveillance and prognosis prediction of ESCC. Our report also points to the need for further studies about the role of anti-cancer drug resistance and radiation insensitivity of GSTM3 in ESCC.

## Conclusions

GSTM3 expression may serve as an independent indicator for disease-free survival. Low GSTM3 expression correlated with poor disease-free survival of ESCC.

## Data Availability

All data generated or analyzed during this study are included in this published article.

## Abbreviations

95% CI: 95% confidence interval
DFS: Disease-free survival
DSS: Disease-specific survival
ESCC: Esophageal squamous cell carcinoma
GSTM3: Glutathione S-transferase mu 3
HR: Hazard ratio
IHC: Immunohistochemical
qRT-PCR: Quantitative real-time polymerase chain reaction
ROC: Receiver operative characteristic
TMA: Tissue microarray
